# Trajectory Planning Method for a Robotic Arm Based on an Improved Multi-Objective Golden Jackal Optimization Algorithm

**DOI:** 10.3390/s26092696

**Published:** 2026-04-27

**Authors:** Juan Wei, Jiangle Wang, Manzhi Yang, Bin Feng

**Affiliations:** School of Mechanical Engineering, Xi’an University of Science and Technology, Xi’an 710001, China

**Keywords:** robotic arm, trajectory planning, multi-objective optimization, golden jackal optimization, 3-5-3 piecewise polynomial interpolation

## Abstract

To address the complex challenge of simultaneously optimizing the operation time, motion impact, and energy consumption in industrial robotic arm trajectory planning, this study proposes a novel multi-objective optimization framework based on an improved multi-objective golden jackal optimization (IMGJO) algorithm. Firstly, the original single-objective Golden Jackal Optimization is extended into a multi-objective formulation by integrating an external Pareto archive and a crowding distance sorting mechanism. This extension effectively generates a well-distributed and highly convergent Pareto-optimal solution set. Secondly, to enhance global exploration capabilities and improve convergence stability, the escape energy model is refined. This is achieved through the synergistic integration of three key strategies: tent chaotic mapping for enhancing the initial population diversity, opposition-based learning to accelerate the early-stage search process, and an elitism preservation strategy to prevent premature convergence and mitigate the risk of entrapment in local optima. Thirdly, the IMGJO algorithm is integrated with a 3-5-3 polynomial interpolation scheme to establish a kinematically constrained trajectory planning model, ensuring a generation of smooth, continuous, and dynamically feasible joint space trajectories. Finally, comprehensive comparative experiments against several state-of-the-art benchmark algorithms demonstrate that the proposed IMGJO framework significantly outperforms its counterparts in terms of both convergence speed and the quality of the Pareto solution set. Furthermore, experimental validation on the Yaskawa HP-20D robotic arm platform demonstrates that the proposed method can effectively achieve a comprehensive optimization of execution time, impact, and energy consumption. Compared with the pre-optimization trajectory, the total operation time is reduced by 2.42%; the impacts of Joint 1 and Joint 2 are reduced by 74.65% and 75.82%, respectively; and the energy consumption of Joint 1 and Joint 2 are reduced by 27.11% and 26.83%, respectively. Moreover, the generated trajectory is smooth and continuous, thereby significantly improving the operational efficiency and stability of the robotic arm.

## 1. Introduction

With the rapid advancement of modern manufacturing, the performance requirements for industrial robotic arms are becoming increasingly stringent [1]. Consequently, trajectory planning methods based solely on a single objective have proven insufficient to meet the comprehensive demands of real-world applications, which necessitate a trade-off between the operation time, motion smoothness, and energy consumption. Early research efforts primarily concentrated on optimizing the individual performance metrics. For example, Wenhua Nan et al. [2] utilized the modification/target algorithm, while Qiongyang Wen et al. [3] applied a pyramid topology particle swarm optimization algorithm to minimize either trajectory execution time or energy consumption. Similarly, Peng [4] performed global optimization with the primary goal of smoothing joint acceleration profiles. However, as operational scenarios and task constraints become more complex, the limitations of single-objective optimization in balancing the overall system performance have become apparent. This has led to a gradual shift towards multi-objective optimization as the dominant paradigm. Recent studies have made significant progress in this area: Jingkai Cui et al. [5] and Ronghua Liu et al. [6] employed the improved Grey Wolf Optimizer and Cuckoo Search algorithms, respectively, to achieve a simultaneous optimization of the operation time and motion impact. Subsequently, Jiahui Wang et al. [7] and Feng Xu et al. [8] further incorporated energy consumption as a third critical objective, leveraging the improved multi-objective PSO and NSGA-II to conduct collaborative optimization. These multi-objective approaches effectively compensate for the inherent limitations of their single-objective counterparts, offering a more holistic strategy for enhancing the operational efficiency and task adaptability of a robotic arm.

The Golden Jackal Optimization (GJO) algorithm, proposed by Chopra et al. [9] in 2022, is a nature-inspired metaheuristic that mathematically models the cooperative hunting strategies of golden jackals to perform global search and optimization within a defined solution space. Compared to the other established optimization techniques, such as the artificial bee colony algorithm and multi-objective particle swarm optimization (MOPSO), GJO offers distinct advantages, including a minimal number of control parameters, a structurally simple framework, and a cooperative search mechanism that is inherently suitable for addressing the multi-joint coordination challenges that are prevalent in robotic manipulator control. Nevertheless, despite its potential, the standard GJO exhibits several notable limitations in practical applications, such as insufficient population diversity, suboptimal convergence accuracy, and a high susceptibility to premature convergence and entrapment in local optima [10]. To overcome these drawbacks, numerous enhancement strategies have been proposed. For instance, Panliang Yuan et al. [11] developed a hybrid approach incorporating dynamic lens imaging learning and a golden sine mechanism to bolster global exploration and accelerate convergence. Similarly, Shijie Jiang et al. [12] integrated sine–cosine and Cauchy mutation operators, significantly improving the algorithm’s efficacy in solving complex unmanned aerial vehicle path planning problems. Furthermore, modified versions of GJO have demonstrated considerable promise in diverse fields, including engineering optimization, structural design [13], and machine learning [14]. However, it is crucial to note that the majority of these existing improvements are typically designed for specific optimization tasks. They often lack the robustness required to address the unique and demanding characteristics of industrial robotic arm trajectory planning, which necessitates a sophisticated multi-objective optimization framework, strict kinematic and dynamic constraints, and high-precision solutions. Consequently, there remains a clear need for targeted algorithmic enhancements to specifically adapt GJO to this complex application domain.

In summary, this study addresses the complex problem of multi-objective trajectory planning for industrial robotic arms by developing a comprehensive optimization framework. Initially, forward and inverse kinematic models of the robotic arm are established, upon which a multi-objective cost function is formulated to concurrently minimize the operation time, motion impact, and energy consumption. To ensure the physical feasibility of the generated paths, a 3-5-3 segmented polynomial interpolation model is constructed to represent continuous and smooth joint space trajectories. Given the inherent limitations of the standard Golden Jackal Optimization (GJO) algorithm in handling multi-objective optimization problems, the multi-objective golden jackal optimization (MGJO) is first proposed. This is achieved by integrating a Pareto elite archive and a crowding distance mechanism, which enhances the algorithm’s ability to manage constraints and generate a well-distributed set of non-dominated solutions. However, the basic MGJO still faces challenges related to premature convergence and suboptimal population diversity. To overcome these deficiencies, the present work further refines the escape energy model and incorporates three synergistic strategies: the tent chaotic map to enhance initial population diversity, opposition-based learning to expedite early-stage convergence, and an elite retention strategy to prevent stagnation at local optima. This culminates in the development of the Improved multi-objective golden jackal optimization algorithm, designed to achieve superior global search capability and convergence accuracy. The final IMGJO framework is then integrated with the 3-5-3 polynomial interpolation model for trajectory planning in joint space. For a fair and comprehensive evaluation of the trade-offs between conflicting objectives, min–max normalization is employed to map all objectives into a dimensionless space. Systematic simulation studies and physical experiments conducted on a Yaskawa HP-20D robotic arm prototype validate the proposed method, demonstrating its effectiveness in achieving a balanced optimization among time, impact, and energy, and confirming its strong engineering feasibility.

Compared with existing studies, this paper makes the following main contributions: (1) a three-objective trajectory planning model for a robotic arm is developed by jointly considering execution time, motion impact, and energy consumption. In combination with a 3-5-3 piecewise polynomial interpolation scheme, the proposed model ensures the continuity and smoothness of the planned trajectory; (2) to overcome the limitation that the original GJO algorithm is only applicable to single-objective optimization problems, a Pareto archive and a crowding distance mechanism are incorporated to develop a MGJO, thereby enabling it to solve constrained multi-objective trajectory planning problems; and (3) to address the issues of premature convergence and insufficient population diversity in MGJO, an improved version of MGJO, termed IMGJO, is further proposed by refining the escape energy model and integrating tent chaotic mapping, opposition-based learning, and an elite retention strategy, thereby enhancing the algorithm’s global search capability and convergence accuracy.

## 2. Kinematics Analysis

**Figure 1:**
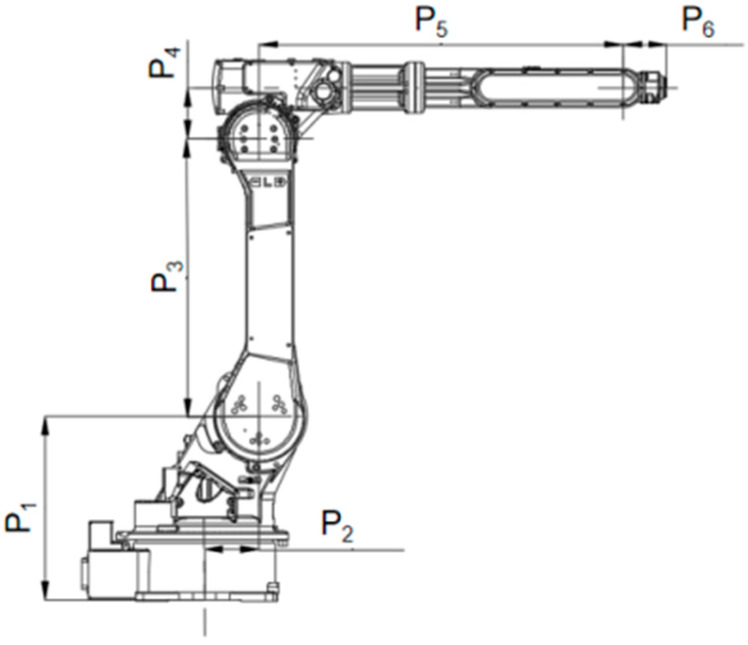
The Yaskawa HP-20D robotic arm.

The Yaskawa HP-20D industrial manipulator shown in Figure 1 is employed as the experimental object in this study. Its main structural link lengths are as follows: *P*_1_ = 0.505 m; *P*_2_ = 0.105 m; *P*_3_ = 0.760 m; *P*_4_ = 0.140 m; *P*_5_ = 0.795 m; *P*_6_ = 0.105 m.

**Figure 2:**
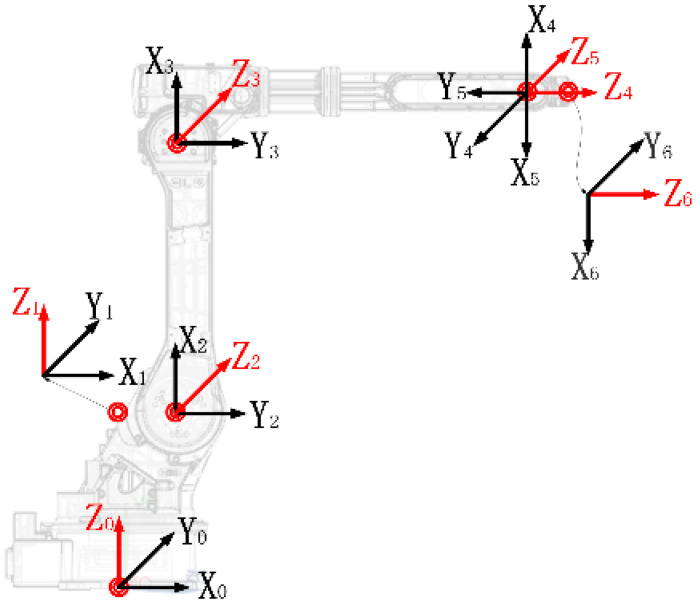
A distribution diagram of link coordinate systems.

For the kinematic analysis of the robotic arm, this study employs the Denavit–Hartenberg (D-H) convention to establish a mathematical model of the manipulator under investigation. Figure 2 illustrates the joint coordinate systems constructed using this methodology. Table 1 summarizes the assigned D-H parameters required for modeling, including the link twist angle (*α*_i_), link length (*a*_i_), joint angle (*θ*_i_), and link offset (*d*_i_) for each link, where *i* = 1, 2, …, 6.

### 2.1. Forward Kinematics Analysis

Based on the geometric parameters of the robotic arm and the relative poses between adjacent link coordinate systems, a homogeneous transformation matrix from coordinate system {*i* − 1} to {*i*} can be constructed. In accordance with the modified Denavit–Hartenberg (D–H) convention, the homogeneous transformation matrix Tii−1 of link *i* can be expressed as:

(1) Tii−1=cosθi-sinθi0ai−1sinθicosαi−1cosθicosαi−1-sinαi−1-disinαi−1sinθisinαi−1cosθisinαi−1cosαi−1dicosαi−10001

Substitute the D-H parameters listed in Table 1 into Equation (1) and multiply the homogeneous transformation matrices of each joint successively. Then, the homogeneous pose matrix of the end effector of the manipulator with respect to the base coordinate system can be obtained:

(2) T60=T10T21T32T43T54T65=nxoxaxpxnyoyaypynzozazpz0001

In the formula, Tii−1 represents the homogeneous transformation matrix from coordinate system i − 1 to coordinate system i; the elements *n_x_*, *n_y_*, *n_z_*, *…*, *a_x_*, *a_y_*, *a_z_* in the rotation matrix are used to represent the pose of the end coordinate system {6} relative to the base coordinate system {0}; the displacement components *p*_x_, *p*_y_, *p*_z_ indicate the spatial position coordinates of the origin of the end coordinate system {6} in the base coordinate system {0}.

### 2.2. Inverse Kinematic Analysis

The inverse kinematics problem aims to determine the joint variables of a robotic arm based on the pose parameters of the end effector. In this section, the analytical method is adopted to derive the joint angle parameters of this robotic arm. From the forward kinematics Equation (2), it is known that the direction cosine components *n_x_*, *n_y_*, *n_z_*, *…*, *p*_x_, *p*_y_ and *p*_z_ in the end effector pose matrix are all known quantities; however, the specific values of the link homogeneous transformation matrices T10, T21, T32, T43, T54, T65 are determined by the joint variables, and all joint variables are unknown in the inverse solution process. Therefore, by separating the variables and simultaneously left-multiplying both sides of T60=T10T21T32T43T54T65 by T10−1, we obtain:

(3) T10−1T60=T21T32T43T54T65

Based on the above derivation, the analytical expressions of the joint variables *θ*_1_ to *θ*_6_ can ultimately be obtained. The analytical solution of the inverse kinematics can be expressed as:

(4) $$\theta_{1}=arctan\frac{p_{y}-d_{6}a_{y}}{p_{x}-d_{6}a_{x}}$$

(5) $$\theta_{4}=arcsin(\frac{a_{x}p_{y}-a_{y}p_{x}}{Zs_{5}})$$

(6) $$\theta_{5}=arccos(a_{z}c_{23}-Ss_{23})$$

(7) $$\theta_{3}=arcsin(a_{3}K_{1}-a_{2}s_{23}+K_{2}s_{23})$$

(8) $$\theta_{2}=\theta_{23}-\theta_{3}\;$$

(9) $$\theta_{6}=arctan(\frac{K_{n}}{K_{o}})$$

In the formula, $S=a_{x}c_{1}+a_{y}s_{1}$; $K_{1}=d_{6}c_{5}-d_{4}$; $K_{2}=p_{x}c_{1}+p_{y}s_{1}-p_{z}+d_{1}$; $Z=\sqrt{{a_{x}}^{2}{d_{6}}^{2}-2a_{x}d_{6}p_{x}+{a_{y}}^{2}{d_{6}}^{2}-2a_{y}d_{6}p_{y}+{p_{x}}^{2}+{p_{y}}^{2}}$; $K_{n}=n_{z}c_{23}-(n_{x}c_{1}+n_{y}s_{1})s_{23}$; $K_{o}=o_{z}c_{23}-(o_{x}c_{1}+o_{y}s_{1})s_{23}$; $s_{23}=\sin(\theta_{2}+\theta_{3})$; $c_{23}=\cos(\theta_{2}+\theta_{3})$; $s_{1}=\sin\theta_{1}$; $c_{1}=\cos\theta_{1}$.

### 2.3. Objective Function

To achieve coordinated optimization of the manipulator trajectory with respect to execution time, motion impact, and energy consumption, the time parameters of each segment of the 3-5-3 piecewise polynomial interpolation are selected as the optimization variables, and the decision vector is defined as follows:

(10) $$x={\left[t_{1},t_{2},t_{3}\right]}^{T}$$

In the formula, *t*_1_, *t*_2_ and *t*_3_ denote the durations of the three trajectory segments, respectively. Given the positions of the start point, intermediate point, and end point, as well as the boundary constraints on velocity and acceleration, the coefficients of the 3-5-3 piecewise polynomial can be uniquely determined from *x*, thereby yielding the trajectory of the *m*-th joint over the interval [0, *T*(x)]. The total execution time is defined as follows:

(11) $$T(x)=\sum_{s=1}^{3}t_{s}$$

In the above equation, *t*_s_ denotes the time parameter of the *s*-th trajectory segment.

To achieve coordinated optimization of trajectory planning with respect to the execution time, motion impact, and energy consumption, three objective functions are defined in this study. Specifically, *f*_T_(*x*) represents the total execution time of the manipulator, reflecting the time required to complete the task; *f*_J_(*x*) denotes the total motion impact during the manipulator motion and is used to evaluate joint fatigue and motion smoothness; and *f*_E_(*x*) represents the total energy consumption of the manipulator, indicating the efficiency of energy utilization during the motion cycle. Moreover, the energy consumption that is adopted in this study is an acceleration-based surrogate metric rather than a rigorous physics-based actuator energy model. Accordingly, the “energy consumption” reported in this paper should be regarded as an approximate indicator of energy-related performance rather than a direct measure of actual actuator energy consumption. Their mathematical expressions are given as follows:

(12) $$f_{T}(x)=T(x)=\sum_{s=1}^{3}t_{s}$$

(13) $$f_{J}(x)=\sum_{m=1}^{M}\left(\int_{0}^{T\left(x\right)}{\left({\dddot{\theta }}_{m}(x)\right)}^{2}dt\right)$$

(14) $$f_{E}(x)=\sum_{m=1}^{M}\left(\frac{1}{T(x)}\int_{0}^{T\left(x\right)}{\left({\ddot{\theta }}_{m}(x)\right)}^{2}dt\right)$$

In the above equations, M denotes the number of joints of the manipulator; ${\ddot{\theta }}_{m}(x)$ represents the angular acceleration of the *m*-th joint; and ${\dddot{\theta }}_{m}(x)$ represents the angular motion impact of the *m*-th joint.

Therefore, the multi-objective optimization model for manipulator trajectory planning can be formulated as follows:

(15) $$\underset{x\in \delta }{\min F(x)}=[f_{T}(t),f_{J}(t),f_{E}(t)]$$

In the above formulation, *δ* denotes the feasible region subject to various trajectory constraints, including the joint angle limits, the joint angular velocity limits, the joint angular acceleration limits, and the time constraints for each segment. It is defined as follows:

(16) $$\delta =\left\{\begin{array}{c} \theta_{m}^{\min}⩽\theta_{m}⩽\theta_{m}^{\max}\\ {\dot{\theta }}_{m}^{\min}⩽{\dot{\theta }}_{m}⩽{\dot{\theta }}_{m}^{\max}\\ {\ddot{\theta }}_{m}^{\min}⩽{\ddot{\theta }}_{m}⩽{\ddot{\theta }}_{m}^{\max}\\ t_{s}^{\min}⩽t_{s}⩽t_{s}^{\max} \end{array}\right.$$

In the above equations, $\theta_{m}^{\min}$ and $\theta_{m}^{\max}$ represent the upper and lower bounds of the *m*-th joint angle, respectively; ${\dot{\theta }}_{m}^{\min}$ and ${\dot{\theta }}_{m}^{\max}$ represent the upper and lower bounds of the *m*-th joint angular velocity, respectively; ${\ddot{\theta }}_{m}^{\min}$ and ${\ddot{\theta }}_{m}^{\max}$ represent the upper and lower bounds of the *m*-th joint angular acceleration, respectively; and $t_{s}^{\min}$ and $t_{s}^{\max}$ represent the upper and lower bounds of each time segment, respectively.

Therefore, this study aims to determine the Pareto-optimal solution set with respect to execution time, motion impact, and energy consumption, subject to kinematic and time boundary constraints, thereby generating manipulator trajectories that achieve a balance between efficiency and smoothness.

### 2.4. 3-5-3 Piecewise Polynomial Interpolation

Currently, in the joint space trajectory planning of robotic arms, polynomial interpolation methods are widely adopted [15]. Polynomial interpolation can generate relatively smooth motion trajectories, but there is a trade-off in the selection of polynomial order: an order that is too low may lead to discontinuous acceleration, thereby affecting the smoothness of motion; an order that is too high will significantly increase computational complexity and may introduce numerical oscillations and other issues [16]. To ensure the continuity of displacement, velocity, and acceleration while considering computational efficiency, this paper adopts a 3-5-3 piecewise polynomial interpolation method. The expressions of each piecewise polynomial are as follows:

(17) $$\theta_{i1}(t)=a_{10}+a_{11}t+a_{12}t^{2}+a_{13}t^{3}$$

(18) $$\theta_{i2}(t)=a_{20}+a_{21}t+a_{22}t^{2}+a_{23}t^{3}+a_{24}t^{4}+a_{25}t^{5}$$

(19) $$\theta_{i3}(t)=a_{30}+a_{31}t+a_{32}t^{2}+a_{33}t^{3}$$

**Figure 3:**
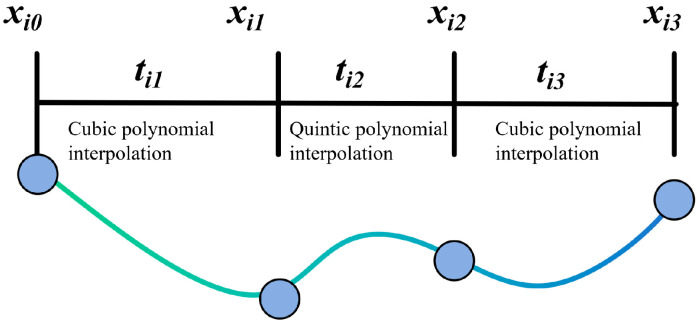
A schematic diagram of 3-5-3 polynomial interpolation.

In the formula, *t* represents the time variable; *θ*_i1_, *θ*_i2_ and *θ*_i3_ denote the angular displacement functions of the *i*-th joint of the robotic arm in the first, second, and third trajectory segments, respectively; and *a*_10_ to *a*_33_ are the undetermined coefficients of each segment’s polynomial. The illustration of the 3-5-3 piecewise polynomial interpolation is shown in Figure 3, where *t*_i1_, *t*_i2_, and *t*_i3_ are the operation times of each trajectory segment; *x*_i0_, *x*_i1_, *x*_i2_, and *x*_i3_ correspond to the trajectory target positions of the starting point, intermediate point 1, intermediate point 2, and the ending point, respectively.

To ensure the continuity and smoothness of the trajectory, the corresponding kinematic constraints are introduced within the framework of the 3-5-3 piecewise polynomial interpolation. In particular, at the starting and ending points of the trajectory, the joint angular velocity and angular acceleration are constrained to zero to ensure smooth starting and stopping; at the intermediate points along the trajectory, inter-segment connection constraints are imposed, requiring the joint position, angular velocity, and angular acceleration to be continuous and equal at the boundaries between the adjacent trajectory segments. These constraints can be expressed in the following mathematical form:

(20) $$\left\{\begin{array}{cc} \begin{array}{l} \theta_{i1}(0)=x_{i0}\\ \theta_{i2}(0)=\theta_{i1}(t_{i1})=x_{i1}\\ {\dot{\theta }}_{i2}\left(0\right)={\dot{\theta }}_{i1}\left(t_{i1}\right)\\ \theta_{i3}(0)=\theta_{i2}(t_{i2})=x_{i2}\\ {\ddot{\theta }}_{i3}\left(0\right)={\ddot{\theta }}_{i2}\left(t_{i2}\right)\\ {\dot{\theta }}_{i3}\left(t_{i3}\right)=0 \end{array} & \begin{array}{l} {\dot{\theta }}_{i1}\left(0\right)=0\\ {\ddot{\theta }}_{i1}\left(0\right)=0\\ {\ddot{\theta }}_{i2}\left(0\right)={\ddot{\theta }}_{i1}\left(t_{i1}\right)\\ {\dot{\theta }}_{i3}\left(0\right)={\dot{\theta }}_{i2}\left(t_{i2}\right)\\ \theta_{i3}(1)=x_{i3}\\ {\ddot{\theta }}_{i3}\left(t_{i3}\right)=0 \end{array} \end{array}\right.$$

In the equation, $\dot{\theta }$ represents the angular velocity of the joint, and $\ddot{\theta }$ represents the angular acceleration of the joint. The subscripts *i*1, *i*2, and *i*3 correspond to the first, second, and third trajectory segments, respectively. Based on the aforementioned constraint conditions, the problem of determining the undetermined coefficients can be organized into a system of linear equations, and the corresponding coefficient matrix *A* is constructed as follows:

(21) $$A=\left[\begin{array}{cccccccccccccc} t_{1}^{3} & t_{1}^{2} & t_{1} & 1 & 0 & 0 & 0 & 0 & 0 & -1 & 0 & 0 & 0 & 0\\ 3_{1}^{2} & 2t_{1} & 1 & 0 & 0 & 0 & 0 & 0 & -1 & 0 & 0 & 0 & 0 & 0\\ 6t_{1} & 2 & 0 & 0 & 0 & 0 & 0 & -2 & 0 & 0 & 0 & 0 & 0 & 0\\ 0 & 0 & 0 & 0 & t_{2}^{5} & t_{2}^{4} & t_{2}^{3} & t_{2}^{2} & t_{2} & 1 & 0 & 0 & 0 & -1\\ 0 & 0 & 0 & 0 & 5_{2}^{4} & 4t_{2}^{3} & 3t_{2}^{2} & 2t_{2} & 1 & 0 & 0 & 0 & -1 & 0\\ 0 & 0 & 0 & 0 & 20t_{2}^{3} & 12t_{2}^{2} & 6t_{2} & 2 & 0 & 0 & 0 & -2 & 0 & 0\\ 0 & 0 & 0 & 0 & 0 & 0 & 0 & 0 & 0 & 0 & t_{3}^{3} & t_{3}^{2} & t_{3} & 1\\ 0 & 0 & 0 & 0 & 0 & 0 & 0 & 0 & 0 & 0 & 3t_{3}^{2} & 2t^{3} & 1 & 0\\ 0 & 0 & 0 & 0 & 0 & 0 & 0 & 0 & 0 & 0 & 6t_{3} & 2 & 0 & 0\\ 0 & 0 & 0 & 1 & 0 & 0 & 0 & 0 & 0 & 0 & 0 & 0 & 0 & 0\\ 0 & 0 & 1 & 0 & 0 & 0 & 0 & 0 & 0 & 0 & 0 & 0 & 0 & 0\\ 0 & 1 & 0 & 0 & 0 & 0 & 0 & 0 & 0 & 0 & 0 & 0 & 0 & 0\\ 0 & 0 & 0 & 0 & 0 & 0 & 0 & 0 & 0 & 0 & 0 & 0 & 0 & 1\\ 0 & 0 & 0 & 0 & 0 & 0 & 0 & 0 & 0 & 1 & 0 & 0 & 0 & 0 \end{array}\right]$$

The specific form of the coefficient matrix *a* is as follows:

(22) $$a={\left[a_{13},a_{12},a_{11},a_{10},a_{25},a_{24},a_{23},a_{22},a_{21},a_{20},a_{33},a_{32},a_{31},a_{30}\right]}^{T}$$

The specific form of the joint-angle matrix *θ* is as follows:

(23) $$\theta ={\left[0,0,0,0,0,0,x_{i3},0,0,x_{i0},0,0,x_{i2},x_{i1}\right]}^{T}$$

Therefore, the relationship $Aa=\theta_{i}\;$ is established. When the durations of the three trajectory segments are known, the coefficient matrix *a* for the 3-5-3 polynomial interpolation can be obtained.

## 3. Trajectory Planning Based on IMGJO

### 3.1. Principles of GJO

#### 3.1.1. Population Initialization

(1) Initialize the population

(24) $$Y_{0}=Y_{\min}+rand(Y_{\max}-Y_{\min})$$

In the above equation, *Y*_min_ and *Y*_max_ denote the lower and upper bounds of the corresponding solution space, respectively, while rand denotes a random variable following the uniform distribution *U*(0,1).

(2) Initialization of the prey matrix

(25) $$Prey=\left[\begin{array}{cccc} Y_{1,1} & Y_{1,2} & \cdots & Y_{1,d}\\ Y_{2,1} & Y_{2,2} & \cdots & Y_{2,d}\\ \vdots & \vdots & & \vdots \\ Y_{n,1} & Y_{n,2} & \cdots & Y_{n,d} \end{array}\right]$$

In the above equation, *Y*_i,j_ denotes the value of the *j*-th dimension of the *i*-th prey in the population, *n* denotes the total number of prey, and *d* denotes the number of decision variables.

(3) Prey Fitness Matrix

(26) $$F_{{|}_{\mathrm{OA}}}=\left[\begin{array}{c} f(Y_{1,1};Y_{1,2};\cdots ;Y_{1,d})\\ f(Y_{2,1};Y_{2,2};\cdots ;Y_{2,d})\\ \vdots \\ f(Y_{n,1};Y_{n,2};\cdots ;Y_{n,d}) \end{array}\right]$$

In the above equation, *F*_OA_ denotes the fitness values of the stored prey individuals, and *f*(…) denotes the fitness function. In the GJO algorithm, the current best solution and the second-best solution correspond to the male and female golden jackals, respectively. Subsequently, the iterative optimization process simulates two hunting behaviors of golden jackals, namely cooperative search and encircling attack.

#### 3.1.2. Collaborative Search

During the hunting process, male golden jackals and female golden jackals complete the search and tracking in a collaborative manner, with the male individuals playing a leading role and the female individuals following and cooperating under their guidance. The position updates of both are regulated by dynamic factors such as the energy of the prey’s escape and are adaptively adjusted with the iterative process. The above-mentioned position update mechanism can be expressed in the following mathematical form:

(27) $$Y_{1}(t)=Y_{M}(t)-E\times |Y_{M}(t)-R_{L}\times Prey(t)|$$

(28) $$Y_{2}(t)=Y_{\mathrm{FM}}(t)-E\times |Y_{\mathrm{FM}}(t)-R_{L}\times Prey(t)|$$

In the formula, *t* represents the current iteration number. *Y*_1_(*t*) and *Y*_2_(*t*) respectively denote the prey positions inferred from the cooperative positions of the male and female golden jackals after the *t*-th iteration update. *Y*_M_(*t*) and *Y*_FM_(*t*) respectively represent the current positions of the male and female golden jackals at the *t*-th iteration, where the female’s position update is guided and influenced by that of the male. *E* is a dynamic control parameter representing the energy of the prey’s escape. *R*_L_ is a random vector that follows a *Lévy* distribution and is used to model random perturbations during the search process. *Prey(t)* represents the prey’s position vector at the *t*-th iteration.

The prey escape energy *E* represents the ability of the prey to escape when being pursued by the golden jackals. The algorithm dynamically adjusts it based on the search status during the iterative process. When |*E*| ≥ 1, it indicates that the prey has a strong defense and escape ability, and the golden jackal population will stop the current pursuit and enter the global exploration stage to expand the search range and find new potential targets; when |*E*| < 1, it means that the prey’s escape energy is insufficient and it is difficult to escape from the hunting range, and the algorithm transitions from exploration to local exploitation, intensifying the search and attack around the current prey position to accelerate convergence.

(29) $$E=E_{1}\times E_{0}$$

(30) $$E_{0}=2r-1$$

(31) $$E_{1}=c_{1}\times \frac{1-t}{T}$$

In the formula, *E*_0_ represents the initial value of the prey’s escape energy; *r* is any value within the range of [0, 1]; *E*_1_ is the prey’s energy that decreases with the number of iterations, and its value shows an increasing trend as the iterations progress; *T* is the maximum number of iterations; *c*_1_ is a constant with a value of 1.5, used to adjust the search intensity and convergence speed of the algorithm.

The *Lévy* distribution can be expressed in the following form:

(32) $$R_{L}=0.05LF(y)$$

(33) $$LF(y)=0.01\times (\mu \times \sigma )/\left|v^{1/\beta }\right|$$

(34) $$\sigma =(\frac{\Gamma (1+\beta )\times \sin(\pi \beta /2)}{\Gamma \left(\frac{1+\beta }{2}\right)\times \beta \times 2^{\left(\frac{\beta -1}{2}\right)}}{)}^{\frac{1}{\beta }}$$

In the above equation, *LF*(*y*) denotes the *Lévy* flight function; *μ* and *v* are random variables that follow a uniform distribution over the interval [0, 1]; and *β* is a constant equal to 1.5.

Additionally, the update strategy for the prey’s position adopts the mean of the results that were calculated by Equations (27) and (28) to generate the estimated prey position for the next iteration.

(35) $$Y\left(t+1\right)=\frac{Y_{1}\left(t\right)+Y_{2}\left(t\right)}{2}$$

#### 3.1.3. Encirclement and Attack

During the pursuit of prey by golden jackals, the escape energy of the prey usually shows a rapid decline. The male and female individuals jointly compress the prey’s evasive space through coordinated tracking and position linkage strategies, thereby gradually building an effective encirclement structure. Once the encirclement is formed, the population immediately enters the attack phase and captures the prey under the continuous local exploitation effect. The above process can be described by the following mathematical model:

(36) $$Y_{1}(t)=Y_{M}(t)-E\times |R_{L}\times Y_{M}(t)-Prey(t)|$$

(37) $$Y_{2}(t)=Y_{\mathrm{FM}}(t)-E\times |R_{L}\times Y_{\mathrm{FM}}(t)-Prey(t)|$$

### 3.2. Improved Multi-Objective Golden Jackal Optimization

#### 3.2.1. Multi-Objective Golden Jackal Optimization

GJO was initially designed for single-objective optimization problems. However, in trajectory planning scenarios, it is often necessary to simultaneously balance multiple performance indicators such as operation time, joint impact, and energy consumption, thus posing the requirements for a multi-objective collaborative optimization of the algorithm. Since GJO has difficulty directly obtaining the Pareto optimal solution set that satisfies the multi-objective constraints, this paper proposes a multi-objective golden jackal optimization (MGJO) based on it. This algorithm borrows the multi-objective particle swarm optimization (MOPSO) idea from the Pareto archive mechanism [17], thereby enhancing the applicability and solution ability of GJO in multi-objective optimization problems. Specifically, MGJO first uses non-dominated sorting to stratify the population individuals, screens out the Pareto optimal solutions of the current iteration, and stores them in a dedicated Pareto archive. Given the limited capacity of the archive, the following situations may occur during the storage and update of the solution set:

(1) When a newly generated solution outperforms a certain solution in the archive in terms of performance, the dominated old solution is deleted, and the new solution is incorporated into the Pareto archive.

(2) When the newly generated solution is not dominated by any solution in the archive and vice versa, the new solution is directly appended to the archive to expand the current set of non-dominated solutions.

(3) When the Pareto archive reaches the preset capacity limit, the crowding distance criterion is used to screen the individuals in the archive. The solutions with larger crowding distances are prioritized for retention, while the redundant solutions with smaller crowding distances are eliminated to free up storage space and maintain the distribution balance and solution set diversity of the Pareto front.

In the individual selection stage of the algorithm, in order to maintain the distribution diversity of the solutions among individuals in the same non-dominated level, this paper introduces the crowding distance metric to further distinguish and sort the individuals [18]. For non-boundary individuals, the calculation formula of their crowding distance is as follows:

(38) $$\left[d_{i}=d_{i}+\left(\frac{f_{i+1}^{m}-f_{i-1}^{m}}{f_{max}^{m}-f_{min}^{m}}\right)\right]$$

In the formula, *d_i_* represents the crowding distance of the *i*-th individual; $f_{m+1}^{m}$ and $f_{m-1}^{m}$ respectively denote the objective function values of the individuals adjacent to the *i*-th individual on the *m*-th objective function; $f_{max}^{m}$ and $f_{min}^{m}$ are the maximum and minimum values of the current population on the *m*-th objective function, which are used for normalizing the crowding distance.

Although MGJO has been extended from single-objective optimization to multi-objective optimization through the introduction of the Pareto archive and the crowding distance mechanism, its population update strategy still follows that of the standard GJO. Therefore, when applied to complex constrained trajectory planning problems, MGJO still exhibits the following shortcomings: (1) the initial population is generated randomly, which may lead to an uneven distribution of individuals and insufficient coverage of the search space; (2) the position update process still depends on the original search mechanism, making the algorithm prone to premature convergence and entrapment in local optima in the middle and later stages; (3) the linear escape energy model struggles to achieve a balance between global exploration in the early stage and local exploitation in the later stage; and (4) high-quality non-dominated solutions obtained in the current generation may be replaced during population evolution, thereby reducing the stability of the Pareto front. To address these issues, this study improves MGJO from four perspectives: initialization quality, search space expansion, dynamic balancing of exploration and exploitation, and the preservation of elite solutions. The resulting algorithm is termed IMGJO.

#### 3.2.2. Improved Evasion Energy Model

One of the core mechanisms of GJO is the energy evasion model, which achieves adaptive regulation of the algorithm’s global exploration and local exploitation capabilities by depicting the attenuation law of the prey’s energy over the iterative process. The standard GJO updates the energy using the linearly decreasing strategy shown in Equation (31). To further enhance the dynamic balance effect of exploration and exploitation, this paper introduces a dynamic adjustment index n in the improved scheme to nonlinearly regulate the energy attenuation process, thereby enhancing the search adaptability of the algorithm at different iterative stages and improving the overall optimization efficiency and convergence accuracy on this basis.

(39) n=1.5+0.0015t

(40) $$E_{1}=c_{1}\times {\left(1-\frac{t}{T}\right)}^{n}$$

In the formula, *n* is the dynamic index, which is used to control the decay rate of *E*_1_.

**Figure 4:**
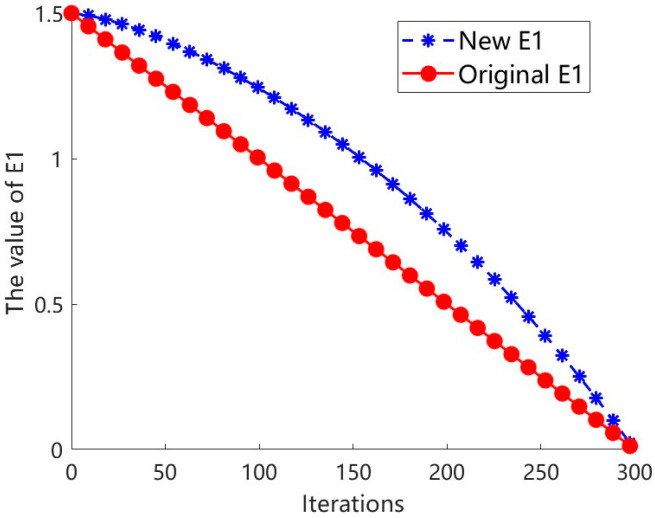
The curve of the E1 value changes.

**Figure 5:**
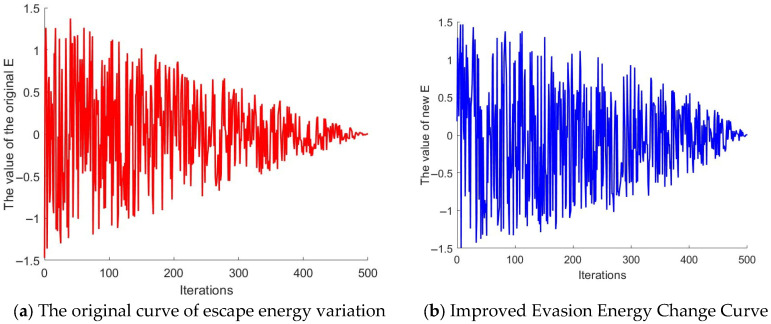
The numerical change curve of evasion energy.

The dynamic variation characteristics of the reduced prey energy *E*_1_ after improvement are shown in Figure 4. It exhibits a clear nonlinear decay pattern with respect to the iterations. Specifically, in the early to middle stages of the algorithm’s operation, *E*_1_ decreases relatively slowly, which helps maintain a strong global exploration ability; while in the later stages of the iterations, the decay rate of *E*_1_ significantly accelerates, thereby enhancing the local development and fine optimization capabilities. Moreover, the comparison results in Figure 5 indicate that the improved escape energy *E* still maintains certain exploration characteristics after approximately 250 iterations; in contrast, the escape energy *E* before improvement entered the stage dominated by local development approximately 150 iterations later. Thus, this improved mechanism effectively extends the algorithm’s global search window, improves the phased allocation of exploration and development, and thereby enhances the overall optimization performance of the algorithm.

#### 3.2.3. Tent Chaotic Map

As GJO generates the initial golden jackal population randomly, it is prone to causing an uneven distribution of individuals in the search space, thereby weakening the comprehensiveness and stability of subsequent searches. To alleviate this issue, this paper introduces the tent chaotic mapping strategy in the population initialization stage, which endows the initialization process with stronger traversal ability and sensitivity to initial values, and to some extent reduces the risk of premature convergence of the algorithm [19]. By reconstructing the distribution of the initial population through this strategy, the uniformity and coverage of the group in the search space can be enhanced. Specifically, a chaotic sequence *Z*_k+1_ is first generated, and based on this, the initial population *Y*_0_ is constructed within the search space, with the expression as follows:

(41) $$Z_{k+1}=\left\{\begin{array}{c} 2Z_{k},\\ 2(1-Z_{k}), \end{array}\right.\begin{array}{c} Z_{k}<0.5\\ Z_{k}\geq 0.5 \end{array},k=1,2,3,\ldots ,N$$

(42) $$Y_{0}=Y_{\min}+Z_{K+1}\times (Y_{\max}-Y_{\min})$$

In the formula, *Z*_k_ represents the chaotic sequence value obtained in the *k*-th iteration and in *Z_k_* ∈ [0, 1]; *Z*_k+1_ represents the sequence value corresponding to the *k*+1-th iteration; *k* is the number of iterations; and *Y*_max_ and *Y*_min_ respectively represent the upper and lower bounds of the solution space.

#### 3.2.4. Reverse Learning

As the GJO algorithm initializes the golden jackal population in a random manner, the initial individuals may show local aggregation in the search space, causing the algorithm’s search to mainly focus on a specific area. This reduces the exploration ability for other potential excellent regions and increases the risk of falling into a local optimum. To alleviate the above problems, this paper introduces a reverse learning mechanism, which expands the search range by constructing a mirror solution of the current solution. This strategy enables the algorithm to consider the reverse solution while evaluating a candidate solution, thereby effectively enhancing the population diversity and global exploration ability at an equivalent iteration cost, and to some extent, reducing the possibility of premature convergence. The reverse population individual *Y*_0_^*^ is defined as:

(43) $${Y_{0}}^{*}=Y_{\min}+Y_{\max}-Y_{0}$$

In the formula, *Y*_max_ and *Y*_min_ represent the upper and lower bounds of the solution space.

#### 3.2.5. Elite Retention Mechanism

To enhance the stability and distribution diversity of the Pareto solution set, this paper introduces a retention strategy based on an elite archive in the algorithm. Specifically, after each generation of evolution, the non-dominated solutions generated by the current population are merged with the elite individuals in the historical archive, and the merged set is further processed using non-dominated sorting to construct a new elite archive, thereby achieving a continuous update and iterative optimization of the Pareto solution set. Given that the archive capacity has an upper limit, when the number of non-dominated solutions after merging exceeds the preset capacity, this paper sorts and truncates the elite candidate individuals based on the crowding distance, prioritizing the retention of individuals with larger crowding distances, to maintain the uniformity of the Pareto front distribution while ensuring convergence performance. This mechanism can effectively retain the excellent non-dominated solutions obtained in the evolution process of the previous generations, reducing the risk of excellent solutions being eliminated during evolution; at the same time, combined with the crowding distance truncation strategy, a more reasonable balance can be achieved between convergence and diversity.

### 3.3. Trajectory Planning Steps

**Figure 6:**
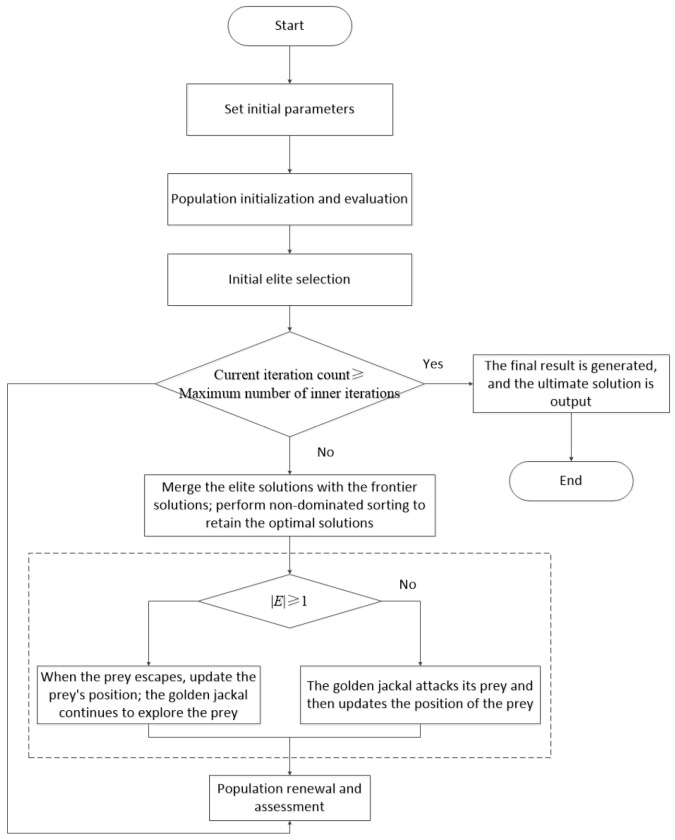
A flowchart of the algorithm.

The overall algorithm flow of IMGJO is shown in Figure 6. When applying this algorithm to the comprehensive optimization problem of runtime, impact and energy consumption, its solution steps can be summarized as follows:

Step 1: Parameter Setting: Set the basic parameters of the algorithm, including the population size *N*, problem dimension, variable upper and lower bounds *Y*_max_ and *Y*_min_, the maximum number of iterations *T*, and the elite rate.

Step 2: Population Initialization and Fitness Evaluation: Generate the initial population using tent chaotic mapping and construct the corresponding reverse population using the reverse learning strategy. Evaluate the fitness of all individuals for the three objectives. Then perform adaptive min–max normalization to update the value ranges of runtime, impact, and energy consumption in real time, ensuring the comparability of objectives of different dimensions during subsequent selection and comparison.

Step 3: Initial Elite Screening and Archive Establishment: Perform non-dominated sorting on the current population to determine the level of each individual on the Pareto front and then calculate the crowding distance to measure the density of the solutions around each individual, thereby maintaining the uniformity of the solution set distribution. Based on the comprehensive evaluation of the Pareto rank and crowding distance, select the top *N* individuals from the candidate set as the next-generation solutions, and initialize the global elite archive.

Step 4: Iteration Termination Judgment: Determine whether the current iteration number has reached the preset upper limit *T*. If the termination condition is met, merge the current population with the historical elite archive and perform non-dominated sorting to obtain the final Pareto optimal solution set for output; otherwise, proceed to the next iteration.

Step 5: Elite Archive Update: Merge the current non-dominated solutions with the elite archive and perform non-dominated sorting, eliminating the dominated inferior solutions and retaining only the high-quality non-dominated individuals. When the archive capacity is exceeded, truncate based on the crowding distance, giving priority to individuals with larger crowding distances to balance the convergence and diversity.

Step 6: Position Update: Calculate the escape energy |*E*| to determine the search state of each individual: when |*E*| ≥ 1, perform an exploration update to expand the search range; when |*E*| < 1, perform a local exploitation update to enhance fine optimization. This generates a new generation of candidate solution sets.

Step 7: Evaluation and Environmental Selection: Based on the new generation population, generate the corresponding reverse population using the reverse learning strategy to enhance the search diversity. Then evaluate the fitness of all solutions and merge them. Determine the superiority and inferiority levels of individuals through non-dominated sorting and assess their diversity using the crowding distance. Finally, select the top *N* individuals with the best performance to form the next-generation population.

## 4. Simulation and Experiment

### 4.1. Experimental Setup and Parameter Settings

#### 4.1.1. Parameter Settings of the Compared Algorithms

This study employs a 3-5-3 piecewise polynomial interpolation method for robotic arm trajectory generation. The performance of the proposed improved multi-objective golden jackal optimization algorithm (IMGJO) is compared with that of the multi-objective golden jackal optimization algorithm (MGJO), the multi-objective particle swarm optimization algorithm (MPSOD) [20], and the hybrid algorithm based on NSGA-II and MOPSO (NMOPSO) [21]. The initial parameter settings of all the algorithms are listed in Table 2. For each joint of the robotic arm, the maximum velocity and acceleration are set to 2.5 rad/s and 2 rad/s^2^, respectively. The initial and target joint configurations are selected from typical handling postures within the reachable workspace of the HP-20D to cover a wide range of motion while avoiding proximity to the joint limits and singular configurations, thereby ensuring the engineering relevance and motion feasibility of the test scenario. Specifically, the initial joint configuration is set to (-pi/3, pi/4, -pi/12, -pi/6, -pi/6, -pi/6), and the target joint configuration is set to (pi/3, -pi/9, -pi/9, pi/9, pi/6, pi/5). All the simulations are conducted in MATLAB R2023a under Windows 11. The computational platform is equipped with an Intel Core i7 processor and 16 GB of RAM.

#### 4.1.2. Sensitivity Analysis of Dynamic Index Parameters

To justify the selection of the dynamic index parameters in Equation (40), a sensitivity analysis was conducted on parameters a and b in n = a + bt. To eliminate the influence of other factors, the population size and the maximum number of iterations were fixed at 100 and 300, respectively, while the remaining algorithmic parameters were kept unchanged during the analysis. Based on the results of the preliminary experiments and the commonly adopted range of nonlinear convergence factors reported in the literature, a was selected from {1.2, 1.5, 1.8}, and b was selected from {0.0010, 0.0015, 0.0020}. Each parameter combination was independently executed 20 times, and the mean value of the comprehensive evaluation index was adopted as the criterion for the overall assessment. The results are presented in Table 3. It can be observed that when a = 1.5 and b = 0.0015, the algorithm achieves the best trade-off among global exploration capability, local exploitation capability, and solution stability, resulting in the optimal comprehensive evaluation index. Therefore, n = 1.5 + 0.0015t was ultimately adopted as the dynamic index expression in the improved escape energy model.

### 4.2. Analysis of the Results of Multi-Objective Trajectory Planning

Due to the fact that multi-objective optimization problems involve requirements such as operation time, energy consumption, and joint impact, and these objectives often have significant differences in magnitude and dimension, if direct optimization and comparison are conducted, it is easy for one objective to dominate the evaluation function, thereby weakening the role of the other objectives and affecting the balance of the Pareto solution set. To eliminate the bias caused by the dimensional differences, this paper adopts the min–max normalization method to map each objective to a dimensionless interval, achieving scale unification among the different objectives. After normalization, the algorithm can more reasonably seek a compromise solution among operation time, energy consumption, and impact, thereby promoting the multi-objective optimization process to discover a more balanced Pareto optimal solution set. Moreover, normalization also simplifies the comprehensive evaluation process of the multi-objective indicators, providing convenience for the subsequent selection of representative optimal solutions based on the ideal point distance criterion. The specific steps are as follows:

(1) Summarize the Pareto front solution sets obtained for each generation. Suppose the front solution set contains multiple non-dominated solutions, each corresponding to three objective function values—operation time, joint impact, and energy consumption. Based on the value range of each objective in this solution set, perform min–max normalization calculations on the operation time, impact, and energy consumption, respectively, to obtain the corresponding dimensionless normalized indicators.

(44) $$T_{i}^{\prime }=\frac{T_{i}-T_{i,min}}{T_{i,max}-T_{i,min}}$$

(45) $$J_{i}^{\prime }=\frac{J_{i}-J_{i,min}}{J_{i,max}-J_{i,min}}$$

(46) $$E_{i}^{\prime }=\frac{E_{i}-E_{i,min}}{E_{i,max}-E_{i,min}}$$

In this formula, $T_{i}$ represents the runtime objective value corresponding to the *i*-th candidate solution in the Pareto solution set. $T_{i,min}$ and $T_{i,max}$ represent, respectively, the minimum and maximum runtime objective values in the current Pareto solution set. ${T_{i}}^{\prime }$ denotes the dimensionless runtime value obtained by min–max normalization. The other symbols are defined in a similar manner.

(2) Search for the ideal solution point

The ideal point can be calculated through the following formula:

(47) $$F_{ideal}=\left(min{T_{i}}^{\prime },min{J_{i}}^{\prime },min{E_{i}}^{\prime }\right)$$

(3) To select representative compromise solutions from the Pareto solution set, this paper calculates the Euclidean distance from the *k*-th solution to the ideal point. The calculation formula is:

(48) $$d_{k}=\sqrt{{\left(T_{k}^{\prime }-T_{ideal}^{\prime }\right)}^{2}+{\left(J_{k}^{\prime }-J_{ideal}^{\prime }\right)}^{2}+{\left(E_{k}^{\prime }-E_{ideal}^{\prime }\right)}^{2}}$$

(4) Ultimately, taking the Euclidean distance $d_{k}$ as a comprehensive evaluation criterion, the candidate solution that minimizes $d_{k}$ is selected from the Pareto solution set, i.e., the trajectory corresponding to the min(*d_k_*) is taken as the overall optimal compromise solution.

**Figure 7:**
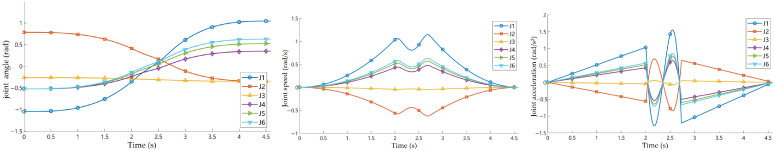
The path curve optimized by MGJO.

**Figure 8:**
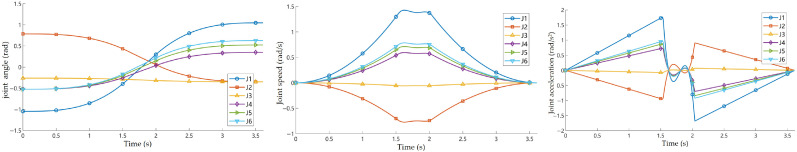
The path curve optimized by IMGJO.

In the context of robotic joint trajectory planning, a smaller value of the evaluation index signifies that the robot can complete the specified task in a shorter duration while simultaneously maintaining lower levels of joint impact and energy consumption. Guided by the aforementioned comprehensive evaluation methodology, 30 independent experiments were conducted using the MGJO algorithm. The optimal evaluation index obtained from these trials was 0.033486, as depicted in Figure 7. Although this result satisfies the fundamental requirements for trajectory planning, it indicates that there remains considerable potential for enhancing the overall performance. Consequently, the MGJO algorithm was improved, leading to the proposal of the IMGJO algorithm. To assess its effectiveness, 30 independent experiments were also performed using IMGJO, yielding an optimal evaluation index of 0.0233. Compared to the MGJO result, this represents a 30.41% reduction in the evaluation index, demonstrating a significant improvement in comprehensive performance. The corresponding experimental results are illustrated in Figure 8.

**Figure 9:**
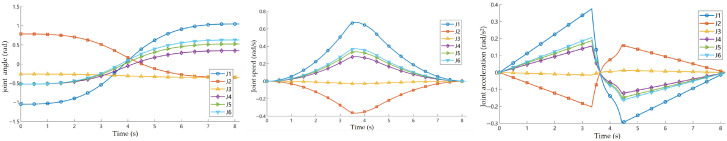
Path Curve Optimized by MPSOD.

**Figure 10:**
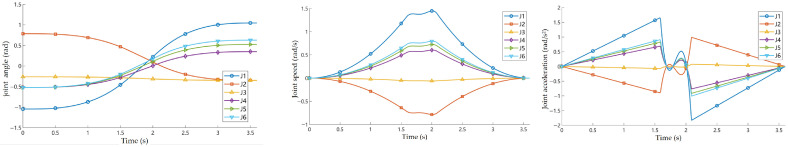
The path curve optimized by NMOPSO.

As illustrated in Figure 7, Figure 8, Figure 9 and Figure 10, a comparative analysis was conducted to evaluate the trajectory optimization performance of the IMGJO, MGJO, MPSOD, and NMOPSO algorithms. Overall, the trajectories generated by IMGJO exhibit the highest quality, characterized by superior smoothness, continuity, and adherence to kinematic constraints. The optimization results of MGJO and NMOPSO are comparable, both outperforming MPSOD and producing trajectories with reasonably good quality. In contrast, MPSOD demonstrates the least effective trajectory optimization performance among the four algorithms evaluated.

**Figure 11:**
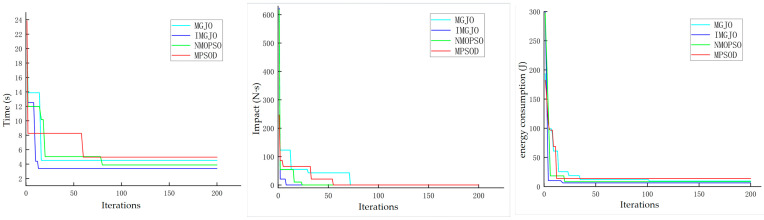
The convergence graphs of time, impact and energy under different algorithms.

Figure 11 illustrates the convergence characteristics of four multi-objective optimization algorithms—IMGJO, MGJO, MPSOD, and NMOPSO—when applied to the trajectory planning of the robotic arm and evaluated across the three objectives of operation time, motion impact, and energy consumption. The results indicate that IMGJO consistently demonstrates the fastest convergence rate and achieves the lowest final objective values across all three metrics, thereby exhibiting a superior comprehensive optimization performance. Specifically, regarding the convergence curve for operation time, IMGJO starts from an initial value of approximately 23 s and rapidly converges within just a few iterations, stabilizing at around 3.6 s, reflecting the highest optimization efficiency. In comparison, MGJO begins with an initial time of roughly 14 s and converges to approximately 5 s. NMOPSO exhibits moderate convergence speed and final values, whereas MPSOD displays the slowest convergence behavior. For the motion impact objective, IMGJO and NMOPSO converge quickly toward zero, followed by MGJO and MPSOD, whose convergence speeds are comparatively slower. Nevertheless, all four algorithms eventually drive the impact index close to zero, confirming their effectiveness in mitigating motion shocks. Regarding energy consumption, IMGJO maintains its superior performance, converging to the lowest energy consumption level within approximately 20 iterations and remaining stable thereafter. MGJO, NMOPSO, and MPSOD exhibit slightly slower convergence rates, with final stabilized values marginally higher than those achieved by IMGJO. In summary, IMGJO shows better convergence behavior than the benchmark algorithms across the three objectives in this test case, suggesting improved optimization efficiency. Its repeated run results further indicate a relatively stable performance with respect to the adopted comprehensive evaluation index. NMOPSO ranks second in overall performance, while MGJO and MPSOD are more susceptible to convergence stagnation during iterative processes and display a greater tendency to become trapped in local optima.

To verify the stability and repeatability of the proposed IMGJO algorithm, IMGJO, MGJO, MPSOD, and NMOPSO were independently executed 20 times each under identical parameter settings. From the final Pareto solution set obtained in each run, the compromise optimal solution was determined using the ideal point distance method, and the best values, mean values, and standard deviations of the evaluation indicators were calculated. The results are presented in Table 4.

As shown in Table 4, IMGJO achieves the lowest mean value and the smallest standard deviation for the adopted comprehensive evaluation index among the compared algorithms. This suggests that IMGJO provides relatively stable results across the repeated independent runs under identical parameter settings. Although MPSOD can also achieve relatively good results in some individual runs, its mean evaluation index and variability are both inferior to those of IMGJO. In contrast, MGJO and NMOPSO exhibit relatively large standard deviations, indicating that they are more sensitive to random initialization and show more pronounced fluctuations across repeated runs.

### 4.3. Pareto Frontier Analysis

**Figure 12:**
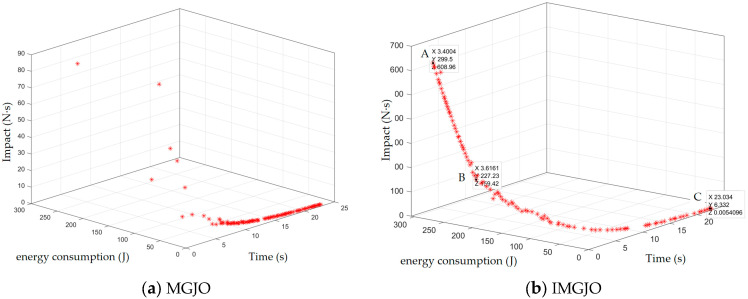
The Pareto frontier.

As illustrated in Figure 12a, the Pareto front generated by the MGJO algorithm exhibits several notable limitations. Specifically, the solution set is prone to stagnation within local optimal regions, resulting in a relatively scattered distribution of front points and insufficient overall convergence. In stark contrast, the Pareto front obtained using the proposed IMGJO algorithm Figure 12b demonstrates markedly superior optimization characteristics. The IMGJO algorithm effectively circumvents the influence of local optima, achieving significant improvements in both the uniformity of the solution set distribution and the precision of the convergence. These visual results provide compelling evidence of the effectiveness of the proposed enhancement strategies in elevating the performance of multi-objective optimization.

To analyze the distribution and trade-off characteristics of the obtained Pareto front, three representative non-dominated solutions were selected and designated as A, B, and C, which were listed sequentially from the top to the bottom of the front. Solution A represents the scheme with the shortest operation time, whereas Solution C achieves the minimum values in both energy consumption and motion impact. Solution B, which exhibits the smallest value for the comprehensive evaluation index, is identified as the most balanced compromise solution across all the performance metrics. As the Pareto front transitions from Solution A to Solution C, a clear trend emerges: the operation time progressively increases, while both energy consumption and motion impact decrease correspondingly. This observation highlights a positive correlation between the energy consumption and the motion impact, as well as a pronounced trade-off relationship between these two objectives and the operation time. The specific objective function values corresponding to these three characteristic solutions are summarized in Table 5.

A comparative evaluation was conducted among candidate solutions A, B, and C to identify the most effective trade-off. As indicated in the results, Solution B exhibits the lowest value for the comprehensive evaluation index, signifying its superior overall performance. Specifically, the trajectory execution time for Solution B is 3.616 s, representing a marginal increase of 6.34% compared to Solution A. However, this slight extension in time yields substantial benefits: Solution B achieves a 24.13% reduction in energy consumption and a 73.82% decrease in motion shock relative to Solution A. Thus, with a relatively minor time investment, the system gains significant improvements in energy efficiency and operational stability. While Solution C demonstrates an even better performance in terms of energy consumption and shock mitigation, these advantages are attained at the cost of a considerably longer operation time. A comprehensive trade-off analysis suggests that Solution C does not constitute a more practical compromise. Therefore, guided by the principle of minimizing the comprehensive evaluation index, Solution B is ultimately selected as the optimal, all-encompassing solution for this study and is adopted as the implementation benchmark for the project.

**Figure 13:**
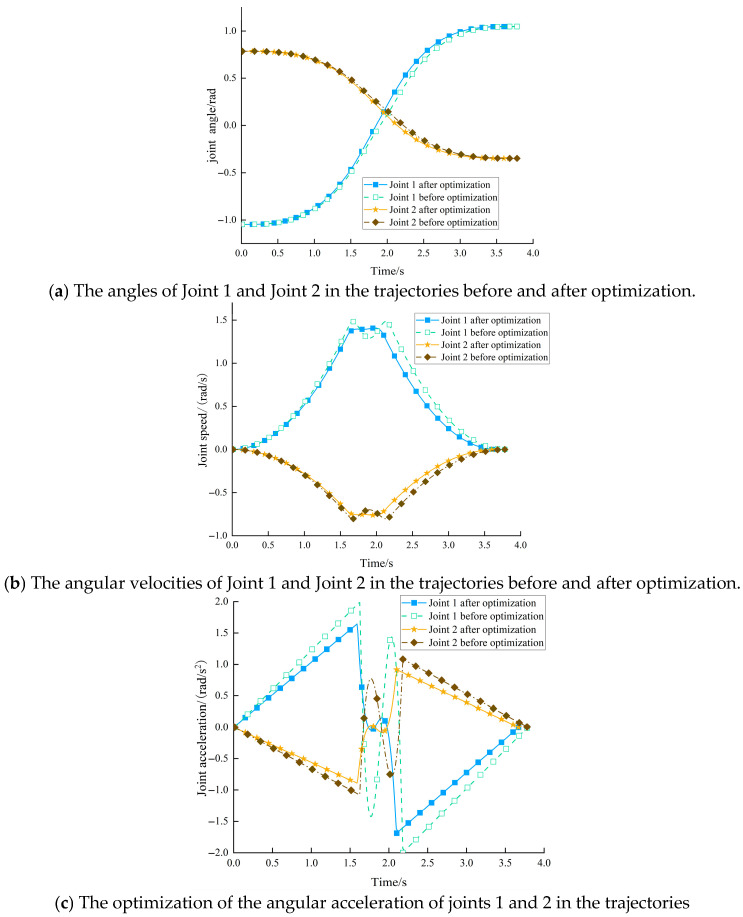
The angles, velocities, and accelerations of the joints before and after optimization.

To evaluate the optimization performance of IMGJO, the pre-optimization trajectory is defined as the baseline trajectory, which is generated without applying any intelligent optimization algorithm. Specifically, given the same starting point, via points, and end point, only 3-5-3 piecewise polynomial interpolation is employed to construct the joint trajectory. The trajectory coefficients are then determined using empirically preset segment time parameters, thereby yielding a baseline trajectory that satisfies the constraints on joint angle, angular velocity, and angular acceleration. A comparative analysis was conducted between the pre-optimization baseline trajectory and the optimized solution B obtained via IMGJO. The angular position, angular velocity, and angular acceleration profiles for the two critical joints are presented in Figure 13. A visual inspection of the figure reveals that the post-optimization curves are markedly smoother, with a significant reduction in the fluctuation range of both angular velocity and angular acceleration. This indicates that the IMGJO algorithm not only achieves a reduction in the total operation time but also effectively suppresses drastic changes in angular acceleration. Consequently, the execution efficiency and operational stability of the robotic arm are substantially improved.

As depicted in Figure 13, the optimized joint trajectory strictly adheres to the predefined kinematic constraints. The resulting trajectory curves are characterized by high continuity and smoothness, exhibiting no discernible discontinuities or abrupt changes throughout the motion sequence. A quantitative assessment, based on the relevant kinematic and dynamic formulas, is presented in Table 6. This table summarizes the calculated impact indices, energy consumption, and the maximum velocity and acceleration for each joint, both before and after the optimization process. The data in Table 6 clearly demonstrate the efficacy of the IMGJO algorithm. Specifically, the optimization yields a 74.65% reduction in motion impact and a 27.11% decrease in energy consumption for Joint 1. Similarly, for Joint 2, the impact is reduced by 75.82%, and the energy consumption is lowered by 26.83%. In conclusion, the IMGJO-based optimization framework not only effectively shortens the total operation time but also significantly mitigates the motion impact and reduces the energy consumption. This leads to a substantial enhancement in the smoothness and overall stability of the robotic arm’s movement, thereby providing a robust and effective solution for high-performance trajectory planning.

### 4.4. Experimental Verification

The physical experimental platform consisted of a six-degree-of-freedom Yaskawa HP-20D industrial manipulator and an NX100 robot controller. The host computer communicated with the controller via Ethernet for program transfer and data management. In the experiments, the robot was operated in a joint space trajectory tracking mode, in which the optimized joint trajectories generated offline were executed by the controller. Specifically, the optimized joint trajectories obtained in MATLAB were first discretized into time-ordered joint reference points at a sampling interval of 0.01 s. These reference points were then converted into executable robot programs using offline programming software and uploaded to the NX100 controller via Ethernet. The controller executed the uploaded trajectory at a sampling frequency of 100 Hz. To ensure repeatability, each experiment started from the same initial joint configuration used in the simulation. The same target trajectory, controller settings, and operating conditions were maintained throughout all the tests. No additional payload or external disturbance was introduced during the repeatability experiments. Under these identical conditions, ten repeated physical experiments were conducted to evaluate the execution repeatability and practical feasibility of the proposed method. During execution, the controller synchronously recorded the actual joint position feedback of all six joints. The recorded data were exported and processed offline in MATLAB. The main preprocessing steps included time-axis alignment between the reference and measured trajectories, unit unification of the joint variables, and consistency checking of the sampled data. The actual end-effector trajectory was then reconstructed through the forward kinematic model based on the measured joint angles.

Let $q_{d}(t)$ and $q_{a}(t)$ denote the desired and actual joint trajectories, respectively. The joint tracking error is defined as:

(49) $$e_{q}(t)=q_{d}(t)-q_{a}(t)$$

The maximum joint tracking error is calculated as:

(50) $$e_{q,\max}=\underset{t}{\max}\underset{i}{\max}\left|q_{d,i}(t)-q_{a,i}(t)\right|$$

Similarly, let $p_{d}(t)$ and $p_{a}(t)$ denote the desired and actual end-effector positions, respectively. The end-effector position error is defined as

(51) $$e_{p}(t)={\left|\left|q_{d,i}(t)-q_{a,i}(t)\right|\right|}_{2}$$

The maximum end-effector error is calculated as

(52) $$e_{p,\max}=\underset{t}{\max}\;e_{p}(t)$$

**Figure 14:**
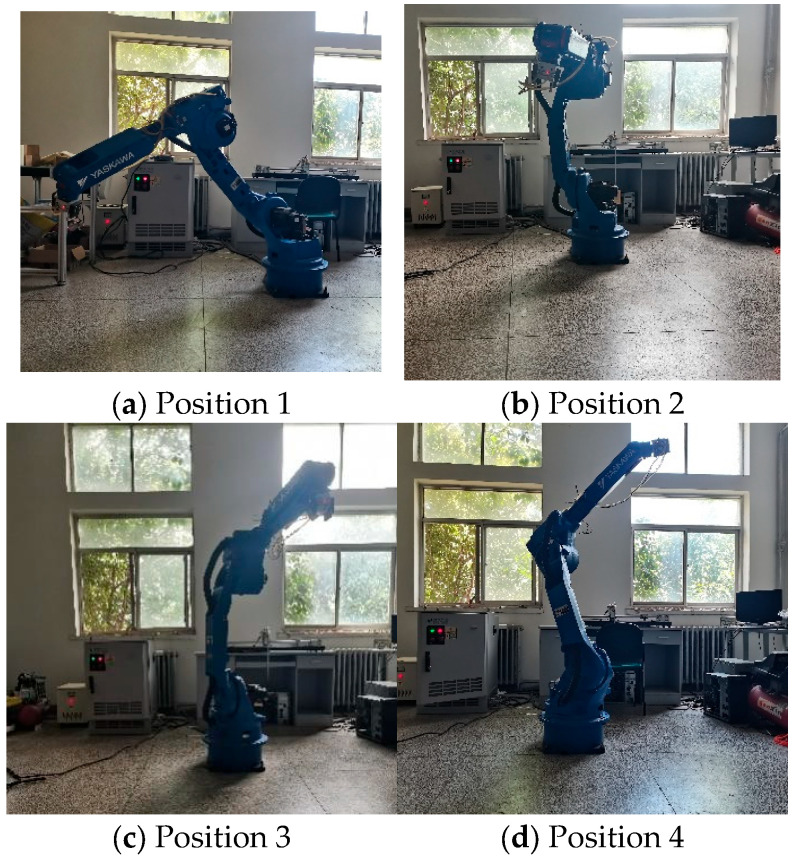
The motion planning trajectory of the robotic arm.

The dynamic variations in the end-effector pose of the robotic arm during operation are shown in Figure 14, and Table 7 lists the representative joint-angle and position data at several sampling instants. The statistical results of the repeatability tests are summarized in Table 8.

A comprehensive analysis of Figure 14 and Table 7 reveals that the Yaskawa HP-20D robotic arm is capable of strictly adhering to the trajectory generated by the IMGJO algorithm, executing the motion plan with high stability. Throughout the entire operational cycle, the displacement, velocity, and acceleration profiles of all joints exhibit continuous and smooth transitions, devoid of any discernible discontinuities, abrupt changes, or oscillatory behavior. These experimental observations confirm that the proposed IMGJO framework can effectively deliver a comprehensively optimized trajectory for industrial robotic arms. The high-fidelity tracking performance demonstrates the method’s exceptional reliability in practical execution and confirms its capacity to ensure superior operational stability.

Furthermore, to verify the stability and repeatability of the proposed algorithm, ten repeated experiments were conducted under identical operating conditions. The corresponding statistical results are presented in Table 8. The results show that the total operation time of the manipulator, the end position error, and the joint tracking error all remain within a narrow fluctuation range, indicating that the proposed method has good repeatability under the tested experimental conditions and shows promising engineering applicability.

## 5. Conclusions

This paper presents a comprehensive trajectory planning methodology for industrial robotic arms, effectively addressing the challenging multi-objective collaborative optimization of the operation time, motion impact, and energy consumption. The proposed framework is based on an IMGJO algorithm. The foundation of the method involves establishing precise forward and inverse kinematic models for the robotic arm and constructing joint space trajectories using a 3-5-3 piecewise polynomial interpolation scheme. A multi-objective cost function is formulated to integrate the three critical performance indicators. To overcome the inherent limitations of the standard GJO in multi-objective scenarios, the algorithm is enhanced by introducing a Pareto elite archive and a crowding distance mechanism, enabling the generation of a uniformly distributed non-dominated solution set. Furthermore, the core escape energy model is refined and synergistically integrated with a tent chaotic map, opposition-based learning, and an elite retention strategy. These enhancements collectively improve population diversity, strengthen global search capabilities, and increase convergence accuracy. The IMGJO algorithm is subsequently coupled with the 3-5-3 interpolation model to perform multi-objective trajectory optimization, with min–max normalization employed to facilitate a unified and comprehensive evaluation of candidate solutions. Simulation results demonstrate that, when benchmarked against NMOPSO, MPSOD, and the basic MGJO, the IMGJO algorithm produces a Pareto front with superior convergence and distribution characteristics. The optimal compromise trajectory derived from this front exhibits significant improvements in operation time, energy consumption, and motion impact. The resulting trajectory curves are demonstrably continuous and smooth, ensuring stable and reliable robotic motion. A detailed quantitative analysis reveals that, compared to the pre-optimization baseline, the motion impact of Joint 1 and Joint 2 was reduced by 74.65% and 75.82%, respectively, while the energy consumption was reduced by 27.11% and 26.83%. Finally, controller-programming verification on a physical Yaskawa HP-20D robotic-arm platform confirmed that the manipulator can stably and accurately track the IMGJO-generated trajectory, validating the method’s engineering feasibility and practical applicability.

In conclusion, the proposed IMGJO-based trajectory planning method provides an effective solution for global optimization and trade-off decision-making in complex robotic systems. It significantly enhances the comprehensive performance and execution stability of trajectory planning, exhibits high engineering applicability, and serves as a valuable reference for intelligent optimization and advanced motion control of an industrial robotic arm.

## Figures and Tables

**Table 1:** D-H parameters table of the manipulator.

Link/*i*	α_i−1_/(rad)	*a*_i−1_/(m)	*d*_i_/(m)	θ_i_/(rad)	Joint Limit/(rad)
1	0	0	0.505	θ_1_	±π
2	−π/2	0.150	0	θ_2_	−π/2~17π/12
3	0	0.760	0	θ_3_	−11π/12~25π/12
4	−π/2	0.140	0.795	θ_4_	±10π/9
5	π/2	0	0	θ_5_	−5π/18~23π/18
6	π/2	0	0.105	θ_6_	±2π

**Table 2:** The parameter settings of the compared algorithms.

Algorithm	Primary Parameters
IMGJO	Elite rate = 0.05, *c*_1_ = 1.5, *β* = 1.5
MGJO	*c*_1_ = 1.5, *β* = 1.5
MPSOD	*c*_1_ = 1.5; *c*_2_ = 2; *w*_max_ = 1.5; *w*_min_ = 1; *P*_m_ = 0.1;
NMOPSO	*c*_1_ = 1.8; *c*_2_ = 2; *w*_max_ = 1.5; *w*_min_ = 0.9; *N*_S_ = 20;

The population size is set to 100, and the maximum number of iterations is set to 300. The lower bound and upper bound of the parameter are set to 0.5 s and 10 s, respectively, and the dimensionality is set to 3; the archive capacity is 100.

**Table 3:** The parameter sensitivity analysis table.

(a, b)	Average Comprehensive Evaluation Index	Standard Deviation
(1.2, 0.0010)	0.0311	0.00102
(1.2, 0.0015)	0.0293	0.000824
(1.2, 0.0020)	0.0242	0.000827
(1.5, 0.0010)	0.0266	0.000665
(1.5, 0.0015)	0.0231	0.000227
(1.5, 0.0020)	0.0264	0.000761
(1.8, 0.0010)	0.0238	0.000465
(1.8, 0.0015)	0.0279	0.000687
(1.8, 0.0020)	0.0288	0.000744

**Table 4:** The statistical results of 20 independent runs for different algorithms.

Algorithm	Best Value	Mean Value	Standard Deviation
IMGJO	0.0227	0.0231	0.000227
MGJO	0.0264	0.0268	0.000405
MPSOD	0.0238	0.0253	0.000281
NMOPSO	0.0760	0.0796	0.000359

**Table 5:** The feature solutions.

Solution	Time t/(s)	Energy Consumption E/(J)	Impact J/(N·s)	Evaluation Index	Solution
A	3.4004	299.5	608.96	0.030017	A
B	3.616	227.23	159.42	0.023382	B
C	23.034	6.332	0.0054	0.688885	C

**Table 6:** The comparison of joint parameters before and after optimization.

Joint	Parameter	Before Optimization	After Optimization	Change Rate/%
Joint 1	*t*/s	3.71	3.62	2.42
	*v*_max_/rad/s	1.485	1.408	5.18
	*a*_max_/rad/s^2^	1.98	1.643	17.02
	*J*_max_/N·s	289.01	73.264	74.65
	*E*_max_/J	260.46	189.849	27.11
Joint 2	*t*/s	3.71	3.62	2.42
	*v*_max_/rad/s	−0.804	−0.763	5.10
	*a*_max_/rad/s^2^	1.083	0.914	15.60
	*J*_max_/N·s	88.902	21.495	75.82
	*E*_max_/J	23.2390	17.003	26.83

**Table 7:** The sequence of joint positions.

Time/s	Joint Angle/rad	Position/m
0	[−1.0472, 0.7854, −0.2618, −0.5236, −0.5236, −0.5236]	[0.7964, −1.3269, 0.7601]
1.56	[−0.3424, 0.4036, −0.2912, −0.2299, −0.1712, −0.1361]	[1.2814, −0.4525, 1.2595]
2.07	[0.3556, 0.0255, −0.3203, 0.0609, 0.1778, 0.2479]	[0.9311, 0.3471, 1.6419]
3.63	[1.0472, −0.3491, −0.3491, 0.3491, 0.5236, 0.6283]	[0.2397, 0.4510, 1.8581]

**Table 8:** The statistical results of repeatability tests in physical experiments.

Metric	Mean	Standard Deviation
Total Execution Time (s)	3.62	0.116
Maximum End-Effector Error (mm)	0.522	0.0305
Maximum Joint Tracking Error (°)	2.86	0.0081

## Data Availability

The data are contained within the article.
